# Regulation of the Immune Checkpoint Indoleamine 2,3-Dioxygenase Expression by Epstein–Barr Virus

**DOI:** 10.3390/biom11121792

**Published:** 2021-11-30

**Authors:** Leila Sawada, Antonio Carlos Rosário Vallinoto, Igor Brasil-Costa

**Affiliations:** 1 Immunology Laboratory, Virology Section, Evandro Chagas Institute, Ananindeua, Pará 67030-000, Brazil; igorcosta@iec.gov.br; 2 Postgraduate Program in Virology (PPGV), Evandro Chagas Institute, Ananindeua, Pará 67030-000, Brazil; 3 Virology Laboratory, Institute of Biological Sciences, Federal University of Pará, Belém 66075-110, Brazil; vallinoto@ufpa.br

**Keywords:** Epstein–Barr virus, immune checkpoints, indoleamine 2,3-dioxygenase

## Abstract

Epstein–Barr virus (EBV) is an oncovirus ubiquitously distributed and associated with different types of cancer. The reason why only a group of infected people develop cancer is still unknown. EBV-associated cancers represent about 1.8% of all cancer deaths worldwide, with more than 150,000 new cases of cancer being reported annually. Since EBV-associated cancers are described as more aggressive and more resistant to the usual treatment compared to EBV-negative ones, the recent introduction of monoclonal antibodies (mAbs) targeting immune checkpoints (ICs) in the treatment of cancer patients represents a possible therapy for EBV-associated diseases. However, the current mAb therapies available still need improvement, since a group of patients do not respond well to treatment. Therefore, the main objective of this review is to summarize the progress made regarding the contribution of EBV infection to the expression of the IC indoleamine 2,3-dioxygenase (IDO) thus far. This IC has the potential to be used as a target in new immune therapies, such as mAbs. We hope that this work helps the development of future immunotherapies, improving the prognosis of EBV-associated cancer patients.

## 1. Introduction

Epstein–Barr (EBV) is the first human oncovirus ever described and known to transform primary B cells in vitro [1,2]. Although it infects approximately 95% of the population worldwide [3], for reasons that are not completely understood, not all of the infected people develop cancer [4]. EBV has a significant health impact since EBV-associated cancers represent about 1.8% of all cancer deaths worldwide, with more than 150,000 new cases of cancer being reported annually [5,6]. Moreover, EBV-associated cancers are usually described as more aggressive and more resistant to the usual treatments [7,8,9,10,11,12]. It is clear that new approaches are necessary in order to improve the prognosis of these patients.

The recent introduction of monoclonal antibodies (mAbs) targeting immune checkpoints (ICs) as a treatment for cancer patients represents a possible therapy for EBV-associated diseases. However, this mAb therapy still needs improvement, since a group of patients showed serious adverse effects and/or did not respond to the actual mAb therapy [13,14,15,16,17,18]. Therefore, our review aims to summarize the progress made regarding the contribution of EBV infection to the expression of the IC indoleamine 2,3-dioxygenase (IDO) so far. In order for a complete understanding of how immunotherapy is a promising treatment for EBV-associated cancers, we also summarize (a) EBV infection, (b) IDO expression in EBV associated-diseases, (c) EBV regulation of IDO, and (d) concomitantly, of an important immune therapy axis, programmed cell death 1 (PD-1)**/** programmed cell death 1 ligand 1 (PD-L1).

## 2. EBV Infection and Its Associated Diseases

### 2.1. EBV Virus

EBV is a double-stranded DNA Gammaherpesvirus composed of a nucleoid, capsid, membrane, and tegument [4,19], with a genome of 172 kb that encodes about 85 genes [19,20]. The virus has been described to infect B lymphocytes, epithelial, and NK cells. The initial steps during infection are not well described in the last two cell types. In B lymphocytes, upon infection, EBV gp350 interacts with complement C3d receptor 2 (CR2), located on the surface of B cells, followed by EBV gp42 binding with major histocompatibility complex (MHC) class II molecules, culminating in the fusion of the virus and cell membrane [9,21,22,23,24]. In CR2-negative epithelial cells, EBV may utilize viral proteins BMRF-2 or gH/gL to attach integrins to the cell surface. Ephrin receptor 2 has been identified as the entry receptor that interacts with viral gH/gL. Interestingly, EBV gp350 and gp42 are non-essential for epithelial cell infection [22,24,25,26].

The virus establishes a lifelong infection, mainly in B lymphocytes and epithelial cells [27]. In these cells, EBV has two types of life cycles: lytic and latent, which are each characterized by the expression of a specific set of genes (Table 1).

### 2.2. EBV Infection and EBV-Associated Tumors

Infection by this oncovirus has been reported to be associated with several types of cancers, such as lymphomas and carcinomas [20,28,29]. Most EBV-associated cancers are lymphomas of B cell origin and carcinomas derived from epithelial cells. Moreover, a group of T/NK cell origin lymphomas is also considered EBV-associated cancer [29]. The closest association between EBV and cancers is seen in undifferentiated nasopharyngeal carcinoma (NPC) and T/NK lymphomas that are 100% EBV positive [29]. It is interesting that each one of the EBV-associated diseases presents a particular profile of viral latency, in which the expression of a set of viral proteins and RNAs is expressed, and other sets are silenced [20,28], demonstrating that EBV expression is elegantly controlled (Table 1).

Some of the EBV-associated cancers are aggressive types, showing resistance to the usual treatments [8,10,11,12]. In fact, EBV-associated cancers are associated with the patient’s immune status [30,31]. For instance, certain EBV-associated malignancies, such as PTLDs, tend to develop in immunocompromised patients [20,28]. In this case, EBV-infected cells show latency III status, which is characterized by the expression of the full spectrum of EBV proteins, which is opposite of what is observed in other EBV-associated cancers that present more restricted forms of latency (latencies II, III, and 0) and are often developed in the absence of such immunosuppression [29]. These factors indicate that the immune response is crucial to EBV-associated cancers.

Multiple strategies of immune escape by this virus have been reported [32]. Most recently, growing evidence shows that EBV proteins have the potential to upregulate ICs [33,34,35], which are important regulators of immune homeostasis and, therefore, can prevent pathologies [36]. Through these mechanisms, but not solely by them, EBV infection could influence the setting of an immune suppressive profile, contributing to cancer development and aggressiveness.

## 3. Targeting ICs

ICs are essential in controlling the immune response. They prevent ligand–receptor engagement, avoiding diseases such as autoimmune ones. Its applicability in the treatment of malignancies such as cancer has greatly improved the survival of patients and has been revolutionizing the field of cancer treatment [14].

The use of mAbs against, at first, Cytotoxic T-lymphocyte protein 4 (CTLA-4) was associated with several adverse effects. Later on, the outcome of patients improved with the emergence of mAbs targeting the axis of PD-1/PD-L1. Currently, there are seven different IC inhibitors that have been approved for the treatment of patients as first- and second-line therapies [13,14,37]; however, there is a subset of individuals that still do not respond to this therapy by presenting de novo and/or adaptive responses [14,15,16,17,18,38]. For instance, treatment targeting anti-PD-1/PD-L1 therapy in certain types of cancers with microsatellite stability shows poor efficiency [39]. Moreover, the recently approved first-line treatment for metastatic non-small cell lung cancer expressing PD-L1 (≥1%) presented a median overall survival of 17.1 months and overall survival rates of 40%. The median duration of response was 23.2 and 6.2 months in chemotherapy patients [40,41]. In melanoma, the combination therapy group which received anti-PD-1 and anti-CTLA-4 mAbs presented a 3-year overall survival rate of 58%. In monoclonal therapy cohorts, rates of 52% and 38% were observed in anti-PD-1 and anti-CTLA-4 groups, respectively [42]. Although these therapies are extremely important and have shaped the treatment of cancer patients in an unprecedented way, the above results indicate the need for the improvement of current mAbs therapies [13] and/or the discovery of other ICs that could be used as targets for immune therapies [36,43].

EBV-associated malignancies have the potential to be specifically treated with IC targeted immune therapy. In fact, several EBV-associated malignancies present higher expression of ICs such as IDO [44] and PD-L1 [45,46,47] when compared to EBV(−) tumor samples. Additionally, in vitro and in vivo reports show a better response to IC blockades in EBV(+) cases [48,49,50,51].

EBV(+) refractory or relapsed non-Hodgkin lymphoma (NHL) patients responded to PD-1 blockade, as opposed to EBV(−) NHL patients [49], and antitumor activity was more evident after PD-1 blockade in EBV(+) DLBCL than in EBV(−) DLBCL cells [50]. A more detailed approach of how EBV is able to regulate the IC PD-L1 is reviewed elsewhere [52].

A group of tumors has the T cell-inflamed tumor phenotype, which is characterized by a large number of tumor-infiltrating lymphocytes (TILs) and interferon (IFN)-transcriptional profiles, including the expression of PD-L1 [53,54], and a high mutation burden responding to checkpoint immunotherapy [55]. On the other hand, the so-called “cold” tumors, because they lack this immune setting, do not respond well to IC-targeting therapy [56]. This possibly indicates that other immunosuppressive mechanisms contribute to the immune-mediated tumor escape and, consequently, tumor regression besides the PD-1/PD-L1 axis. Studies have identified immunosuppressive mechanisms other than the ones directly associated with the PD-1/PD-L1 axis that are present in the T cell–inflamed tumors. These tumors are characterized, for instance, by the presence of Foxp3+Tregs and the tryptophan–kynurenine–aryl hydrocarbon (Trp–Kyn–AhR) pathway [57,58].

## 4. IDO

### 4.1. Trp–Kyn–AhR Pathway

The Trp–Kyn–AhR pathway converts the essential amino acid tryptophan into kynurenine (Kyn) and other secondary metabolites [59]. It is the primary route of tryptophan (Trp) catabolism [60,61]. T cells are extremely sensitive to the local depletion of tryptophan; as kyn binds to AhR, causing T cell differentiation into Tregs, reducing antigen presentation by APCs, and increasing IL-10 production [58,59,62,63,64,65].

IDO-1, by diminishing the availability of Trp and increasing the accumulation of metabolites, also transduces its signaling through the mammalian target of apamycin (mTORC) and general control non-depressible 2 (GNC2). This leads to cell-cycle arrest and/or apoptosis through GCN2 phosphorylation and the inhibition of eukaryotic initiation factor 2α kinase, contributing to the immunosuppressive effects [58,66,67].

IDO-1 is a rate-limiting enzyme that catalyzes tryptophan into Kyn. Later on, it was discovered that IDO-2 is an isoform of IDO-1 and is also able to catalyze Trp; however, functional differences between them have been reported [59,68,69,70]. For instance, it appears that the catalytic activity of IDO-2 is much lower than IDO-1, and IDO2 expression varies according to cancer type [68,71]. Actually, most of the literature does not differentiate between IDO1 and IDO2 [72], and studies are mostly focused on IDO1, so these enzymes will be referred to collectively as IDO unless otherwise specified.

IDO1 is not normally expressed under physiological conditions in human cells, but it is expressed in subsets of antigen-presenting cells (APCs), endothelial, and tumor cells [57,66]. IDO expression can be modulated by other ICs, such as PD-1, PD-L1, and CTLA-4 [73,74,75,76], and cytokines, such as IFN-γ and transforming growth factor-beta (TGF-β), pathogen-associated molecular patterns (PAMPs), damage-associated molecular patterns (DAMPs), and prostaglandin E2 (PGE2) [63,77,78,79,80,81].

Overall, high levels of IDO have been described as a poor prognosis in cancer patients [82,83,84,85,86]. A recent analysis of the Cancer Genome Atlas (TCGA) database indicated that T cell infiltration was more associated with IDO1 than PD-L1, and EBV-associated cancers were correlated with IDO1. Other than that, EBV(+) gastric cancer was also associated with the over-expression of IDO2 [87]. To clarify, among the EBV-associated cancers that were studied, only EBV(+) GC was associated with both IDO2 and IDO1. Overall, EBV-associated cancers usually are associated with the enhanced expression of IDO1 [87]. Either way, this ratifies the fact that EBV(+) gastric cancer is a good candidate for immune therapy treatment, as it has been described as highly immunogenetic [47,88,89]. Interestingly, EBV was not associated with tryptophan-2,3-dioxygenase (TDO) gene expression [87]. TDO encodes an enzyme that is also able to catabolize Trp [90]. Altogether, literature shows that IDO1 might play a key role in EBV-associated cancers.

### 4.2. IDO Expression in EBV-Associated Cancers

IDO is found to be upregulated in several cancer types [66,72,87]. Regarding EBV infection, IDO expression may be induced by EBV in a tumor microenvironment. Cells from the NPC and HL milieu have been described to present stronger IDO expression when compared to tumor cells [33,44]. Importantly, IDO expression was found to be upregulated in EBV-associated HL, particularly in the mixed cellularity subtype [44] and also in EBV(+) oral squamous cell carcinoma when compared to EBV(−) samples [91]. Interestingly, no difference was found regarding IDO expression when the plasmablastic lymphoma (PL) microenvironment from EBV(+) and EBV(−) were compared [45]. This indicates that IDO expression may be correlated with EBV-associated cancers presenting latency II and/or III, since PL is the only cancer of the above-mentioned ones that presents latency I. A summary of the detection of IDO expression in EBV-associated malignancies is in Table 2.

### 4.3. Regulation of IDO Expression by EBV

**Figure 1:**
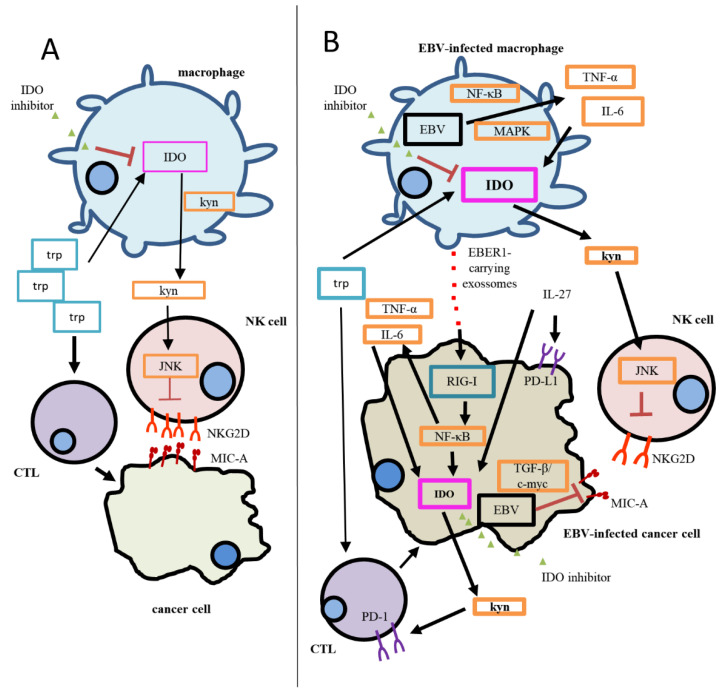
Proposed mechanism of the IDO-specific contribution to immunosuppression in EBV(−) cancer and EBV(+) cancer. (**A**) During homeostasis, CTLs utilize tryptophan in order to eliminate cancer cells. The degradation of tryptophan generates Kyn, which negatively contributes to NKG2D regulation of expression in NK cells. (**B**) EBV has been reported to increase IDO expression in infected cells; therefore, Trp degradation is enhanced, and so is Kyn accumulation. As a result, a more significant immunosuppression status is established, since CTL responses are inhibited, and stronger inhibition of NKG2D in NK cells can be observed. In addition, MIC-A, a NKG2D ligand, has been reported to be downregulated in EBV-infected cancer cells through the TGF-β/c-myc pathway. In this model, the activation of the RIG-I pathway through EBER1, delivered by exosomes, directly regulates IDO expression in cancer cells and/or also activates the NF-kB pathway in them, inducing the production of TNF-α and IL-6, culminating in the upregulation of IDO. The increased IDO expression in cancer cells would exacerbate the immunosuppression by increasing Trp utilization and Kyn accumulation. IL-27 would also play a role in this scenario, contributing to the expression of both IDO and PD-L1; the latter IC receptor expression, PD-1, increases on CD8^+^T cells as Kyn accumulates in the microenvironment. The use of specific IDO inhibitors would have a greater impact on EBV-associated malignancies, since IDO expression is higher when compared to EBV(−) cases. Thick lines represent stronger activation/expression than thin lines (weaker activation/expression). EBV: Epstein–Barr virus; CTL: cytotoxic T cell; JAK3/STAT3: Janus-kinase-3/signal transducer and activator of transcription-3; KYN: kynurenine; MAPK: mitogen-activated protein kinase; MIC-A: MHC class I polypeptide-related sequence A; NF-κB: nuclear factor-κB; NKG2D: NKG2-D type II integral membrane protein; PD-1: programmed cell death protein; PD-L1: programmed death-ligand 1; TGF-β/c-myc: transforming growth factor β/c-myc; TRP: tryptophan.

Reports have shown that EBV infection induces IDO expression. In fact, in vitro infection of primary B cells induced IDO expression [35]. In monocyte-derived macrophages (MDMs), activation of the retinoic acid-inducible gene I (RIG-I) pathway by viral EBER1 delivered from exosomes derived from EBV-infected cells culminated in IDO upregulation through IL-6 and TNF-α (Figure 1). These IDO^+^ cells suppressed the proliferation and cytolytic activity of T cells [91]. Interestingly, EBV infection, through p38 MAPK and NF-kB pathways, increased the production of these cytokines from MDMs, which also resulted in IDO expression [33]. IDO induction did not require IFN-γ, previously known as the main IDO inducer, although it required a synergism between two inflammatory cytokines [33]. This implies that the pathway RIG-I/IL-6/TNF-α/IDO may cross-talk with the MAPK and NF-kB pathways at some point during EBV infection, contributing to IDO expression.

EBER1 is a non-polyadenylated, untranslated RNA, 167 bp in length and is one of the viral RNAs that has been reported to interact with RIG-I. EBERs are known to induce type I interferon production through NF-kB and Interferon regulatory factor 3 (IRF3) [98]. It is interesting to note that the increased expression of RIG-I is observed in EBV(+) cells of cHL of the elderly; however, no differences in the level of IRF3 and IFN-β, two of RIG-I known targets, were detected when compared to EBV(−) samples [99]. This indicates that the recognition of EBER by RIG-I/IL-6/TNF-a is important for IDO expression and, therefore, contributes to an immunosuppressive setting; at the same time, the usual downstream pathway of RIG-I, particularly the IFN production, which is known as a potent antiviral, is affected by several other viral proteins and RNAs at some point during infection. In fact, several reports show the IFN response being hampered directly by EBV [98,100,101].

RIG-I is a cytosolic pathogen-recognition receptor (PRR) that initiates a type-1 interferon (IFN1) response [102,103]. It recognizes short viral double-stranded RNA (dsRNA) and other non-coding RNAs, such as viral RNA and replication transcripts, although it remains difficult to predict exactly which type of motifs and sequences trigger the RIG-I response. Overall, RIG-I has been linked to a response to West Nile virus, Sendai virus, and coronaviruses [102].

EBV, by inducing IDO expression in the infected cell, is able to regulate a series of events in the microenvironment. For instance, EBV-infected MDMexpressingIDO has been shown to impair T cell proliferation and CD8^+^T cytotoxic activity [33]; further, incubation with L-kynurenine, an IDO metabolite, suppresses the JNK pathway in NK cells, reducing NKG2-D type II integral membrane protein and (NKG2D) type II integral membrane protein expression [35]. This activating receptor has been reported to eliminate target cells through granule-dependent cytotoxicity [104]. It also has been implicated in modulating immune responses in important events, such as cancer, viral infections, and chronic inflammatory diseases [105,106]. Interestingly, the EBV-genome-encoded miR-BART7 has been implicated in inhibiting the expression of MHC class I polypeptide-related sequence A, a ligand of NKG2D, partially through the TGF-β/c-myc pathway in NPC cells [106]. Therefore, by upregulating IDO and, consequently, impairing T- and NK-mediated killing, an EBV infection is able to induce a suppressive microenvironment by affecting both innate and adaptive immune responses.

### 4.4. IDO and PD-L1 Regulation

Several reports have pointed to the activation of PD-1/PD-L1 and IDO pathways in cancer samples. In EBV(+) large B-cell lymphoma (LBCL), PD-L1-positive tumors express high levels of IDO [94]. In undifferentiated NPC, significantly increased gene expression of IDO and PD-L1 was observed when compared to normal tissues [96]. Interestingly, it was shown that IFN-γ regulates the expression of both PD-L1, IDO1, and IDO2 through AhR in murine oral cancer cells [107]. In addition, IFN-γ produced by CD8^+^T cells induces the production of Kyn by tumor cells, which induces and activates AhR and upregulates PD-1 expression in CD8^+^T cells [75]. PD-1 is the known receptor of PD-L1 [108].

It seems that IL-27 induces the expression of both IDO and PD-L1 in human cancer cells [109]. Specifically, in cancer cells, IDO1 upregulation is mostly dependent on STAT1, and IL-27 regulation of PD-L1 occurs through signal transducer and activator of transcription 3 (STAT3) [109]. IL-27 is a heterodimer consisting of p28 (IL-27A) and EBV-induced gene 3 (EBI3) chains. EBI3 was found to be upregulated in several B-cell lymphomas and can be regulated by viral LMP1 through NF-κB [110,111]. Indeed, EBI3 has been associated with tumor metastasis and cancer progression [35,112]. In lymphoma cells, EBI3 is expressed without detectable p28 [113,114]. Surrounding macrophages are the probable source of IL-27p28, resulting in the possible upregulation of PD-L1 and PD-L2. It is interesting that the IL-27 heterodimer was able to induce PD-L1/2 expression more efficiently than EBI3 alone [113], meaning that PD-L1/2 expression may be further increased by interaction of EBI3 and macrophage-derived p28.

## 5. Targeting IDO in EBV-Associated Malignancies

Targeting ICs has revolutionized cancer treatment. Although the current available IC-targeting therapies have greatly improved the prognosis of patients [13,115], a significant group of recipients still face serious adverse reactions and/or do not respond to the therapy [116,117,118,119,120,121]; additionally, even the patients who initially respond to mAb therapies may eventually develop a progressive disease with clinical complications [122]. Even though the initial results of preclinical and phase I/II clinical trials with the inhibition of IDO showed promising results [123,124,125,126,127,128,129,130], IDO inhibition during phase III clinical trials has not shown a similar outcome [131]. This may have occurred because of the drug of choice, among other factors that might have influenced the results [132,133]; regardless, new effective treatments with less toxicity are still strongly needed. In this scenario, the IDO and PD-1/PD-L1 cross-talk represents an opportunity for the development of new therapies. Indeed, delivery of siRNAs targeting IDO-1 in mouse DCs has improved the immune response by reducing the number of Treg cells [132]. Other than that, the recent report of a phase I/II clinical trial targeting both of these pathways in melanoma patients is on course through an innovative drug that modulates both PD-L1/PD-1 and IDO pathways and has shown exciting results [134].

In the future, it would also be important to determine during which latency and/or lytic replication programs these phenomena occur, facilitating the development of new target antibodies that could be associated with other current, or soon to be developed, immunotherapies, such as EBV-targeted vaccines. Describing the molecular pathways induced by viral proteins would also be beneficial since they could guide and/or help in the development of new treatments.

In summary, this review showed that the regulation of IDO expression is one of the many mechanisms of immune escape driven by the virus that has the potential of significantly impacting the prognosis of EBV-associated cancer patients. We hope that this review can help with the development and improvement of therapies.

## Figures and Tables

**Table 1:** EBV-associated malignancies and viral latency programs. EBV-associated malignancies show a particular viral latency pattern.

EBV Latency Pattern	Viral Expression Pattern	EBV-Associated Malignancies
III	EBNA-1–6, LMP1, LMP2A-B, EBER, BART	PTLD, DLBCL
II	EBNA-1, LMP1, LMP2A, EBER, BART	HL, NHL, GC, ENKTCL, DLBCL, PBL and NPC
I	EBNA1, EBER, BART	BL, PL
0	EBER, BART	-

Abbreviations: BART: Bam-HI A rightward transcripts; EBER: Epstein–Barrvirus-encoded RNA; EBNA: Epstein–Barr nuclear antigen; LMP: Latent membrane protein; PTLD: Post-transplant lymphoproliferative disorder; DLBCL: Diffuse large B-cell lymphoma; HL: Hodgkin lymphoma; NHL: Non-Hodgkin lymphoma; GC: Gastric cancer; ENKTCL: Extranodal NK/T-cell lymphoma; NPC: Nasopharyngeal carcinoma; BL: Burkitt lymphoma; PL: Plasmablastic lymphoma.

**Table 2:** IDO expression in EBV-associated malignancies.

EBV-Associated Malignancies	IDO Status	Reference
HL	Enhanced when compared to EBV(−)	[44]
DLBCL	Detection/enhanced when compared to EBV(−)	[92,93,94]
EBV(+)GC	Overexpression	[87]
NPC	Detection/overexpression	[33,95,96,97]
PL	Expression was similar to EBV(−)PL	[45]

## Data Availability

Not applicable.
